# Explore autophagy-related lncRNA-miRNA-mRNA ceRNA networks for diagnosis of early-onset schizophrenia through transcriptome analysis

**DOI:** 10.3389/fpsyt.2025.1567148

**Published:** 2025-02-26

**Authors:** Wei Hu, Xinzhe Du, Xinxia Wang, Kexin Zhang, Junxia Li, Yao Gao, Ting An, Hong Zhang, Yu Zhang, Zhiyong Ren, Yong Xu, Sha Liu

**Affiliations:** ^1^ Department of Psychiatry, First Hospital/First Clinical Medical College of Shanxi Medical University, Taiyuan, China; ^2^ Basic Medical College, Shanxi Medical University, Taiyuan, China; ^3^ Shanxi Key Laboratory of Artificial Intelligence Assisted Diagnosis and Treatment for Mental Disorder, First Hospital of Shanxi Medical University, Taiyuan, China; ^4^ Shanxi Province Mental Health Center, Taiyuan Psychiatric Hospital, Taiyuan, China; ^5^ The Eighth Affiliated Hospital, Sun Yat-Sen University, Shenzhen, China

**Keywords:** early-onset schizophrenia, autophagy, long non-coding RNAs, endogenous competitive RNAs, diagnosis

## Abstract

**Background:**

The severe functional impairment and poor prognosis of early-onset schizophrenia (EOS) create a great need to identify effective biomarkers for early diagnosis in young psychiatric patients. Current research indicates a potential link between loss of autophagy function and emotional and behavioral abnormalities in individuals with psychiatric disorders.

**Materials and Methods:**

This study aimed to explore diagnostic autophagy-related endogenous competitive RNA (ceRNA) networks for EOS patients. The messenger RNAs (mRNAs) and long non-coding RNAs (lncRNAs) expression profiles were obtained from peripheral blood mononuclear cells of 18 EOS patients and 12 healthy controls (HC). A co-expression analysis was performed between 365 core lncRNAs and 55 differentially expressed autophagy-related genes (ARGs) to identify differentially expressed autophagy-related lncRNAs. Subsequently, five diagnostic autophagy-related lncRNAs were identified as candidate genes to construct a ceRNA regulatory network using least absolute shrinkage and selection operator (LASSO) Cox regression, and receiver operating characteristic (ROC) curve analysis was performed to evaluate their predictive accuracy. Then, putative interactions among lncRNA-microRNAs (miRNAs)-mRNA were determined based on the lncRNASNP2 and TarBase databases.

**Results:**

Three lncRNAs, twenty miRNAs, and ten mRNAs were selected to construct an autophagy-associated ceRNA network associated with EOS occurrence. Through protein-protein interaction network analysis, five hub mRNAs were identified, which exhibited good predictive ability in distinguishing EOS patients from healthy individuals. ROC curve analysis demonstrated that integrating three diagnostic lncRNAs (RP1-135L22.1, RP5-884C9.2, RP11-390F4.3) along with five hub mRNAs (*EIF4G1*, *AKT1*, *BAX*, *WIPI2*, *MAPT*) appeared to yield better diagnostic accuracy compared to using either lncRNAs or mRNAs alone. Furthermore, all three diagnostic lncRNAs and five hub mRNAs were positively correlated with at least two types of immune infiltration.

**Conclusion:**

Through transcriptome analysis, we searched for diagnostic autophagy-related ceRNA networks, which provided valuable candidates for the early diagnosis of EOS.

## 1 Introduction

Schizophrenia (SCZ) is a devastating mental illness that significantly impacts a substantial portion of the global population (1). SCZ not only affects patients’ mental health but also elevates the risk of physical disorders, leading to a higher loss of life. Compared to the general population, individuals diagnosed with SCZ have a shortened life expectancy of 15-20 years (2). While SCZ typically emerges during late adolescence or early adulthood, its occurrence in childhood and adolescence represents an important subset of psychoses. Early-onset schizophrenia (EOS), characterized by the first appearance of psychotic symptoms before age 18, is considered to have more severe neurodevelopmental abnormalities, a worse clinical course and outcome, a heightened genetic loading, and a greater familial burden of SCZ and associated spectrum disorders than adult-onset SCZ (3). The severe functional impairment and poor prognosis of EOS create a great need to identify effective biomarkers for early diagnosis in young psychiatric patients.

Although many hypotheses related to the etiology of SCZ have been proposed, its neuropathological mechanism has not been established. Cellular autophagy, a unique intracellular degradation process in lysosomes, is essential for neuronal survival and function by maintaining a balance between cell survival and death (4). Current research indicates that SCZ is broadly associated with prominent dysregulation of autophagy function. Studies on autopsy brain tissue of patients with SCZ have shown abnormal expression of key genes involved in regulating neuronal autophagy processes (5). Loss of autophagy function may be related to emotional and behavioral abnormalities in individuals with psychiatric disorders (6). Therefore, identifying biomarkers related to disease progression, prognosis, and treatment responsiveness by exploring the relationship between autophagy and EOS is necessary for early diagnosis and treatment.

Long non-coding RNAs (lncRNAs), a class of non-coding RNAs comprised of more than 200 nucleotides, participate in numerous physiological and pathological processes such as autophagy, apoptosis, and development by regulating transcription, epigenetic modifications, protein translation, etc (7–9). For SCZ, lncRNAs are considered important clues in understanding the molecular mechanisms because they are involved in many steps in the occurrence and development of the disease (10, 11). Currently, endogenous competitive RNAs (ceRNAs) have drawn increasing attention in the field of SCZ. LncRNAs and microRNAs (miRNAs) are important components of the ceRNA networks. LncRNAs act as “molecular sponges”, binding with miRNAs to prevent their inhibitory effect on targeted messenger RNAs (mRNAs). The balance between lncRNAs, miRNAs, and mRNAs can regulate various biological processes, and when disrupted, pathological processes occur (12). The lncRNA-miRNA-mRNA axis is an important regulatory network for SCZ, so they may serve as diagnostic biomarkers for SCZ patients. Current studies have explored the role of autophagy-related ceRNA networks on the diagnosis of SCZ patients, but not all of them included the same lncRNAs/miRNAs/mRNAs, and the accuracy of each model was different (13). In addition, the impact of autophagy-related ceRNAs in EOS has not been clearly defined. Therefore, it is worth exploring diagnostic autophagy-related ceRNAs for EOS patients.

**Figure 1:**
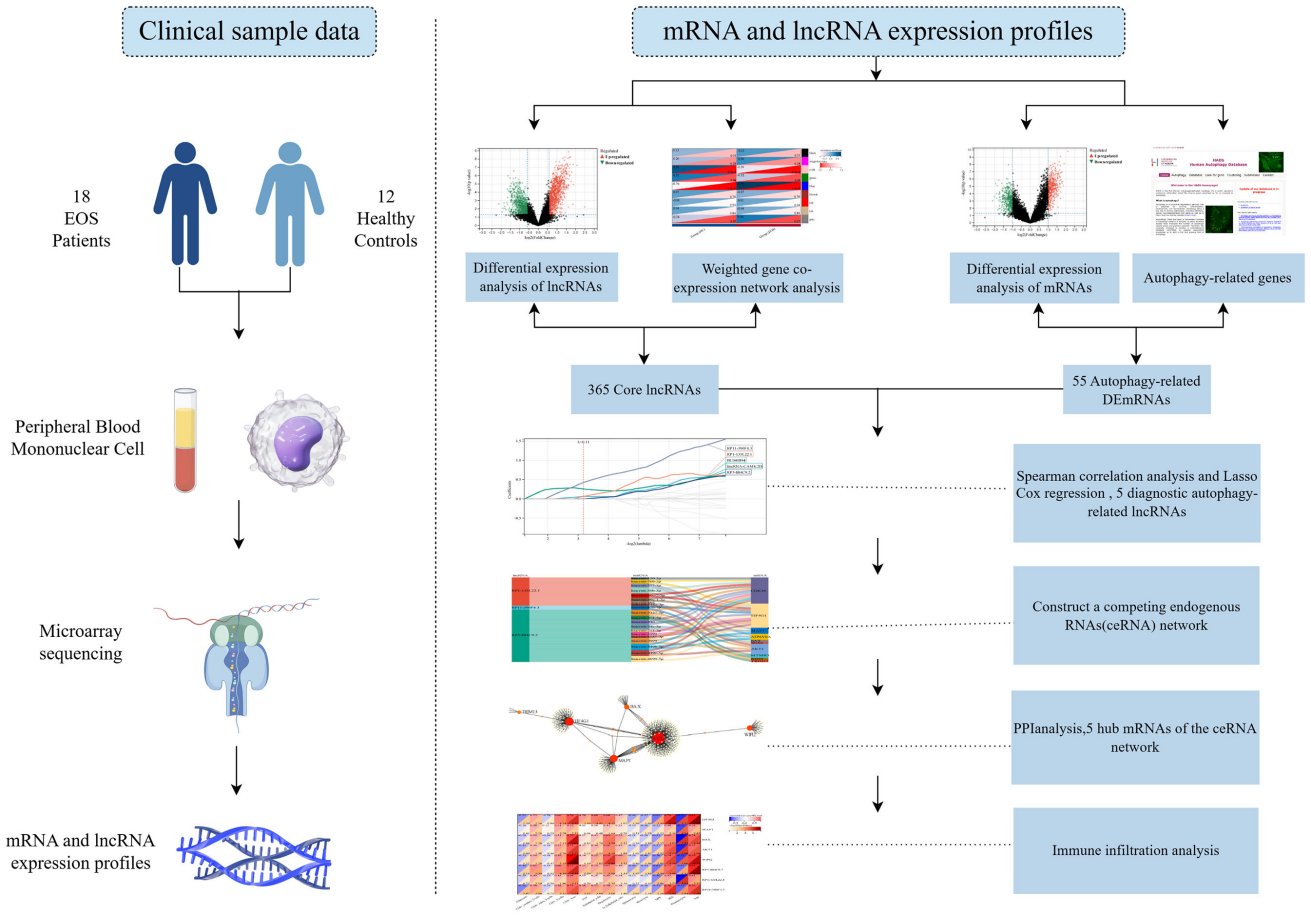
The technical roadmap of this study.

This study aimed to explore diagnostic autophagy-related ceRNA networks for EOS patients. In a first step, we screened 365 core lncRNAs and 55 differentially expressed autophagy-related genes (DEARGs), and identified 365 differentially expressed autophagy-related lncRNAs by a co-expression analysis. Subsequently, five diagnostic autophagy-related lncRNAs were identified as candidate genes to construct a ceRNA regulatory network using least absolute shrinkage and selection operator (LASSO) Cox regression, and receiver operating characteristic (ROC) curve analysis was performed to evaluate their predictive accuracy. Finally, putative interactions among lncRNA-miRNA-mRNA were determined based on the lncRNASNP2 and TarBase databases. **Figure 1** is the technical roadmap of this study (By Figdraw).

## 2 Materials and methods

### 2.1 Data source

The dataset comes from the GSE54913 dataset, which was previously made public by our research group in the Gene Expression Omnibus (GEO) database (https://www.ncbi.nlm.nih.gov/gds). This study recruited 18 EOS patients (10 females and 8 males, aged 14.78 ± 1.70 years) and 12 healthy controls (6 females and 6 males, aged 14.75 ± 2.14 years) (14). We collected peripheral blood mononuclear cell (PBMC) samples from EOS patients and healthy controls. The mRNA and lncRNA expression profiles were obtained using microarrays. All subjects are from the First Hospital of Shanxi Medical University. EOS patients are diagnosed by at least two psychiatrists independently based on the criteria for SCZ in the Diagnosis and Statistical Manual of Mental Disorders Fourth Edition (DSM-IV). The study was approved by the Medical Research Ethics Committee of Shanxi Medical University, and both the subjects and their parents signed informed consent forms.

### 2.2 Identification of differentially expressed mRNAs and lncRNAs

The differential expression analysis was performed via the R package Limma. LncRNAs with a *p*-value < 0.05 and |logFC|> 2, as well as mRNAs with a *p*-value < 0.05 and |logFC|> 1.5, were considered to be statistically significant. The final results were visualized as volcano maps and heat maps using Sangerbox tools (http://sangerbox.com/home.html).

### 2.3 Identification of key trait-module lncRNAs of EOS

The WGCNA (Weighted Correlation Network Analysis) package was used to screen for co-expression modules and key trait-module lncRNAs related to EOS. We chose a soft threshold power *β* = 14 to construct the scale-free topology module. Next, we performed hierarchical clustering to identify co-expression modules, and each module contained at least 100 lncRNAs. Then, we selected co-expression modules that were highly correlated with traits. Pearson’s correlation was used to analyze the module–trait relationships, with a *p*-value < 0.05 defined as a significant correlation. Using gene significance (GS) and module membership (MM), the key trait-module lncRNAs were identified for further analysis. The core lncRNAs of EOS depended on the overlap of differentially expressed lncRNAs (DELs) and key trait-module lncRNAs.

### 2.4 Identification of differentially expressed autophagy-related mRNAs

By intersecting differentially expressed mRNAs (DEGs) with ARGs from the Human Autophagy Database and the GO_AUTOPHAGY gene set in the Gene Set Enrichment Analysis website, we obtained a group of DEARGs. Subsequently, Gene Ontology (GO) function analysis and Kyoto Encyclopedia of Genes and Genomes (KEGG) pathway analysis were used to analyze the biological function of DEARGs.

### 2.5 Identification of differentially expressed autophagy-related lncRNAs

Since the expression data of core lncRNAs and DEARGs did not conform to a normal distribution, we used Spearman correlation to analyze the correlation between them. The lncRNAs with |*r*| > 0.7 and a *p*-value <0.05 were defined as differentially expressed autophagy-related lncRNAs. The interaction relationship network was visualized using Cytoscape software (Version 3.9.1).

### 2.6 Identification of diagnostic-related lncRNAs

We used LASSO Cox regression to screen for autophagy-related lncRNAs with diagnostic value. Then, the predictive accuracy of core lncRNAs was further evaluated using ROC curve analysis.

### 2.7 Construction of the lncRNA-miRNA-mRNA ceRNA network

The lncRNASNP2 database (http://bioinfo.life.hust.edu.cn/lncRNASNP) was utilized to predict the lncRNA-miRNA interaction, while the TarBase v8.0 database (https://microrna.gr/tarbase) was used to predict the targeting miRNAs of DEARGs. The lncRNAs, miRNAs, and mRNAs were visualized as a network using the Sangerbox tools.

### 2.8 Analysis of protein-protein interactions and identification of hub genes

A PPI (Protein-Protein Interaction Networks) network of DEARGs included in the ceRNA network was built using the Search Tool for the Retrieval of Interacting Genes (STRING) database via the Network Analyst v3.0 (https://www.networkanalyst.ca/) web tool, with a confidence score cut-off set at 900. We used degree ≥8 as the screening parameter to discover hub genes. Subsequently, principal component analysis (PCA) was performed to differentiate EOS patients from HC based on the level of hub genes.

### 2.9 Immune infiltration analysis

The xCell algorithm was used to estimate the infiltration abundance of 64 immune and stroma cells in EOS patients and HC. In addition, Pearson correlation analysis was performed to analyze the correlation between hub genes and significantly infiltrated immune cells.

## 3 Results

### 3.1 Identification of DELs and DEGs between EOS and HC

**Figure 2:**
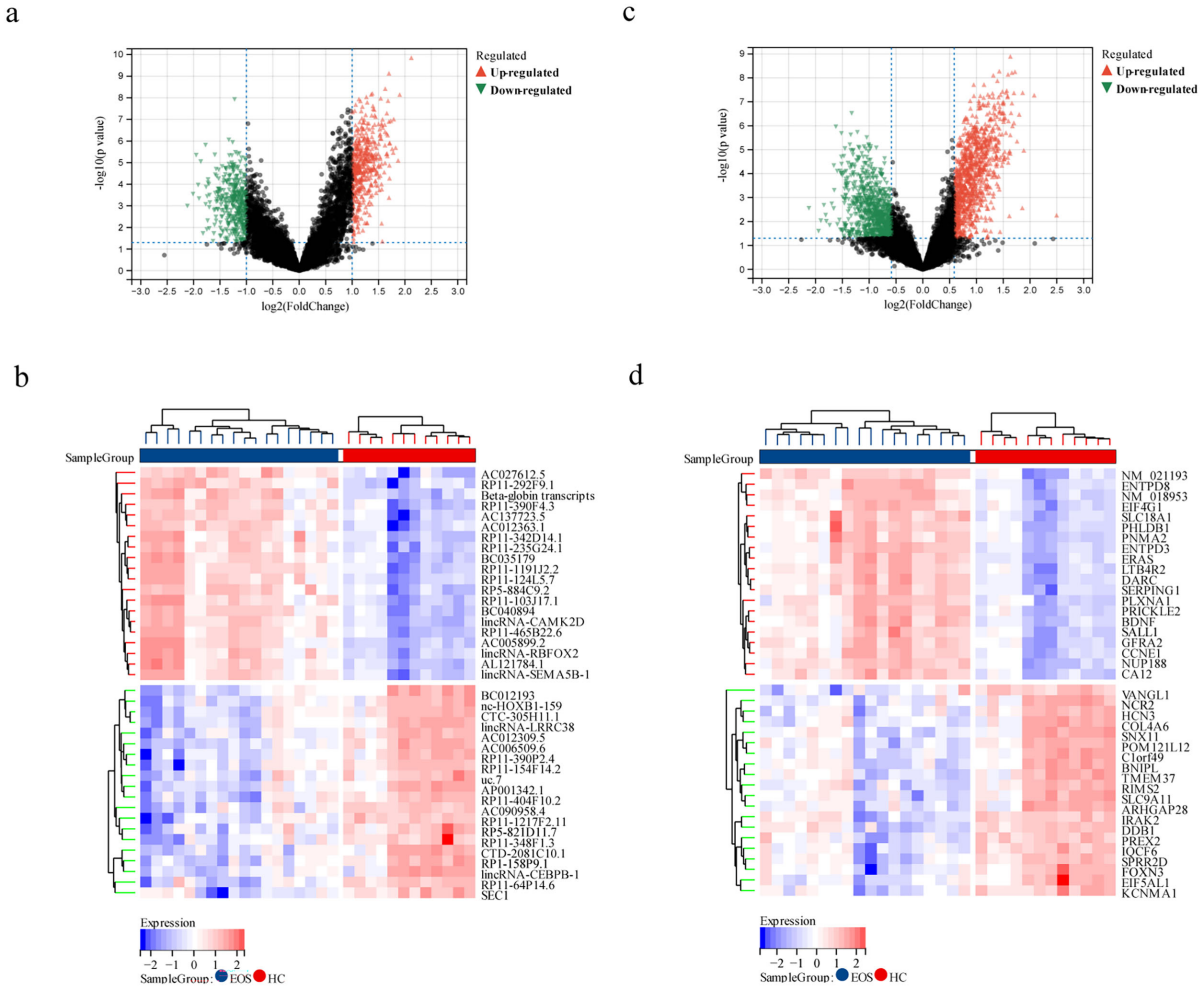
Identification of DELs and DEGs between EOS patients and HC. **(a, b)** Volcano map and heat map depicting DELs between EOS and HC. **(c, d)** Volcano map and heat map illustrating DEGs between EOS and HC.

We initially employed limma differential analysis to screen for DELs and DEGs. A total of 876 DELs and 1929 DEGs were identified in patients with EOS compared to HC. Among these, 461 lncRNAs were upregulated, and 415 were downregulated, while 1033 mRNAs were upregulated, and 896 were downregulated. The volcano plots and heat maps for the DELs and DEGs are presented in **Figures 2a-d**.

### 3.2 Identification of key trait-module lncRNAs

**Figure 3:**
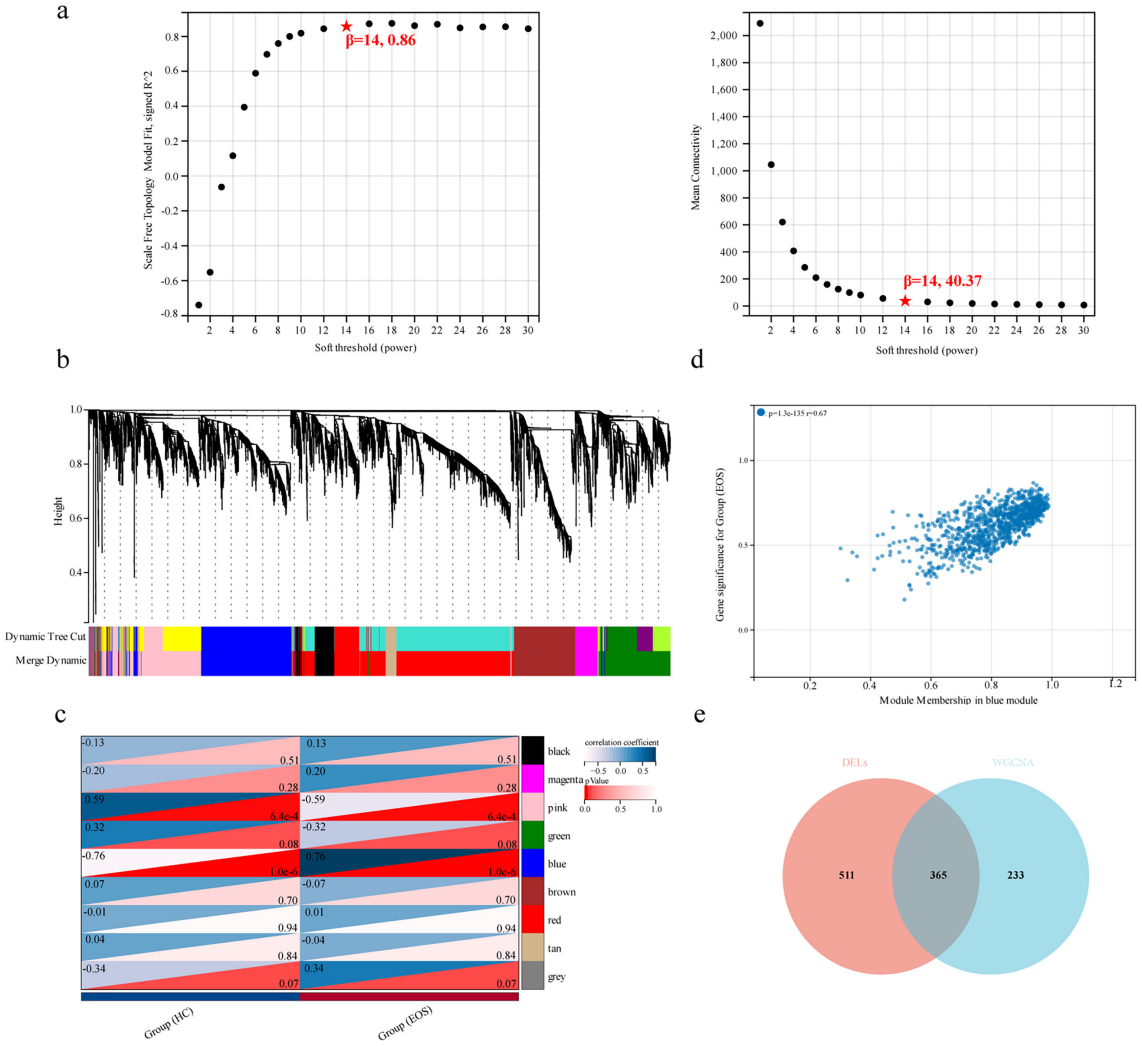
Identification of key trait-module lncRNAs. **(a)** Scale-free index (left panel) and mean connectivity (right panel) of different soft thresholding powers. **(b)** Cluster dendrogram of module eigengenes based on the dissimilarity of topological overlap measure. **(c)** Heatmap showing the correlation between modules and clinical traits. **(d)** Scatter plot of MM and GS for the group (EOS) in the blue module. **(e)** Venn diagram of DELs and key trait-module lncRNAs.

Then, we employed WGCNA to identify characteristic genes within co-expression modules in the lncRNA expression profile. We set the soft threshold power β at 14 to construct the scale-free topology module (**Figure 3a**). A total of 9 modules were identified, and the number of lncRNAs in each module was more than 100. The cluster dendrogram of module eigengenes is shown in **Figure 3b**. The module–trait relationship represented a correlation between modules and clinical traits (**Figure 3c**). The gray module included lncRNAs that did not belong to any module, and the blue module, which has the strongest positive correlation with EOS, was selected as the clinically significant module for further analysis. A scatter plot of MM and GS is shown in **Figure 3d**. Using MM>0.8 and GS>0.1 as thresholds, 598 lncRNAs in the blue module were screened as key trait-module lncRNAs. Finally, 365 core lncRNAs of EOS were selected from the union set between DELs and key trait-module lncRNAs, which was visualized in the Venn diagram (**Figure 3e**).

### 3.3 Identification of differentially expressed autophagy-related mRNAs

**Figure 4:**
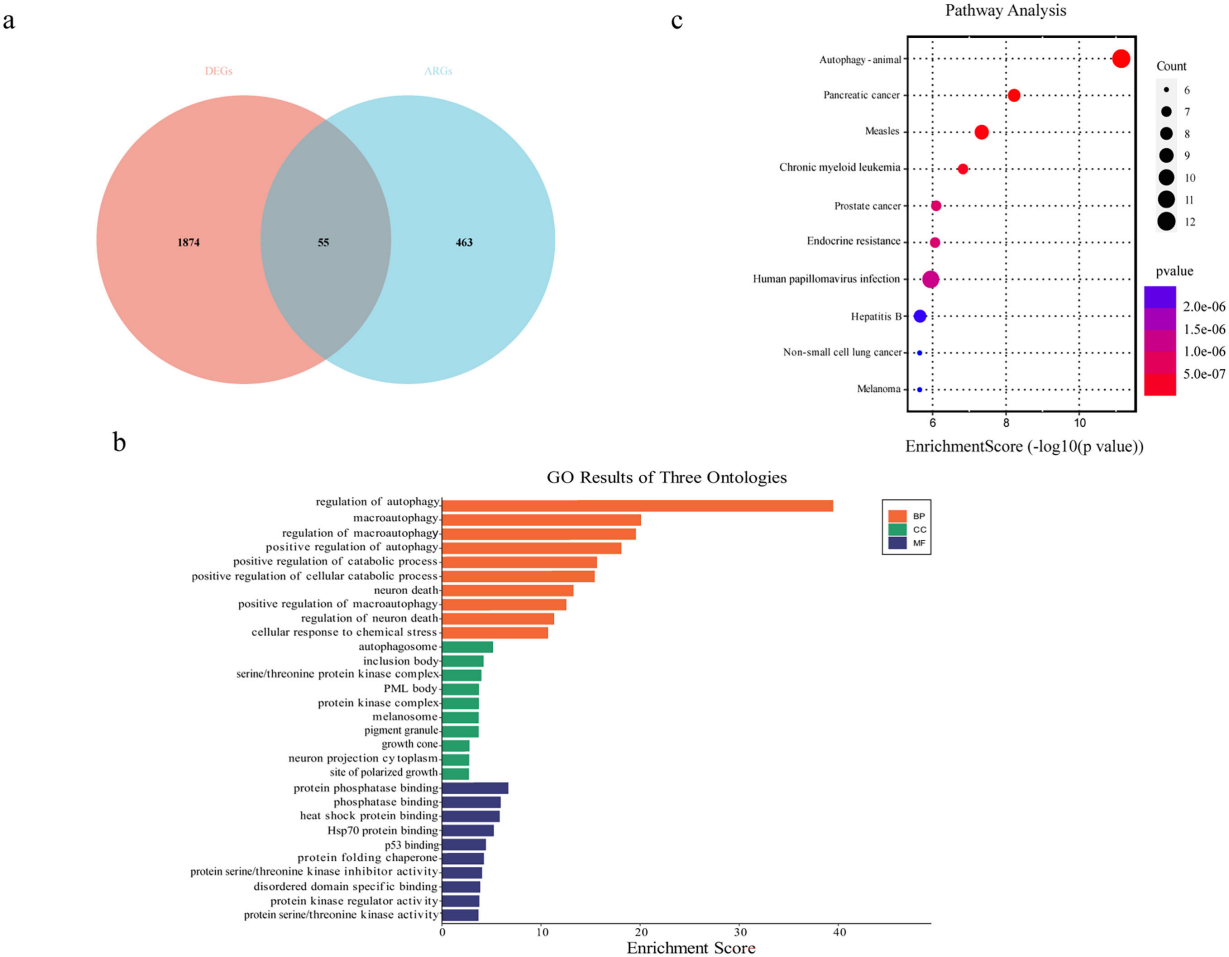
Identification of differentially expressed autophagy-related mRNAs. **(a)** Venn diagram of DEGs and autophagy-related genes. **(b)** GO enrichment analysis of DEARGs. **(c)** KEGG enrichment analysis of DEARGs.

To identify DEGs involved in autophagy, we obtained a list of 518 ARGs from the Human Autophagy Database (http://autophagy.lu/clustering/index.html) and the GO_AUTOPHAGY gene set in the Gene Set Enrichment Analysis website (http://software.broadinstitute.org/gsea/index.jsp). The Venn diagram between ARGs and DEGs was analyzed, and 55 overlapping genes were defined as DEARGs in EOS, including 18 upregulated mRNAs and 37 downregulated mRNAs (**Figure 4a**, **Table 1**). Next, we performed GO enrichment analysis and KEGG pathway analysis of DEARGs. DEARGs were primarily enriched in biological processes such as autophagy and macro-autophagy, as well as cell components such as the autophagosome and inclusion body (**Figure 4b**). They were also enriched in molecular functions such as protein phosphatase binding and heat shock protein binding. Furthermore, these DEARGs were mainly enriched in biological pathways such as autophagy-animal (**Figure 4c**).

**Table 1:** List of autophagy-related genes differentially expressed in EOS.

upregulated	downregulated
*EIF4G1* *AKT1* *ATP6V0A1* *BAX* *WIPI2* *MTMR3* *LRSAM1* *TP73* *ATM* *TSC1* *OPTN* *VHL* *TRIM13* *CDK16* *GFAP* *TRIM34* *MAPT* *SNX6*	*VPS13D* *ATG4C* *ARSB* *ATG16L2* *BAD* *CANX* *CD46* *CDKN1A* *CDKN1B* *CDK5R1* *DAPK1* *DAPK3* *EEF2* *EHMT2* *FAS* *FKBP8* *GRID1* *GSK3A* *HGF* *HSPB1* *HSP90AB1* *IKBKG* *LAMP1* *MAPK8* *MBTPS2* *MTMR4* *NOD2* *PPP1R15A* *PHF23* *PSAP* *RB1* *RALB* *SIRT1* *TMEM49* *TP53INP1* *ZMPSTE24* *RUFY4*

### 3.4 The co-expression network indicated the relationship between diagnostic autophagy-related lncRNAs and DEARGs

**Figure 5:**
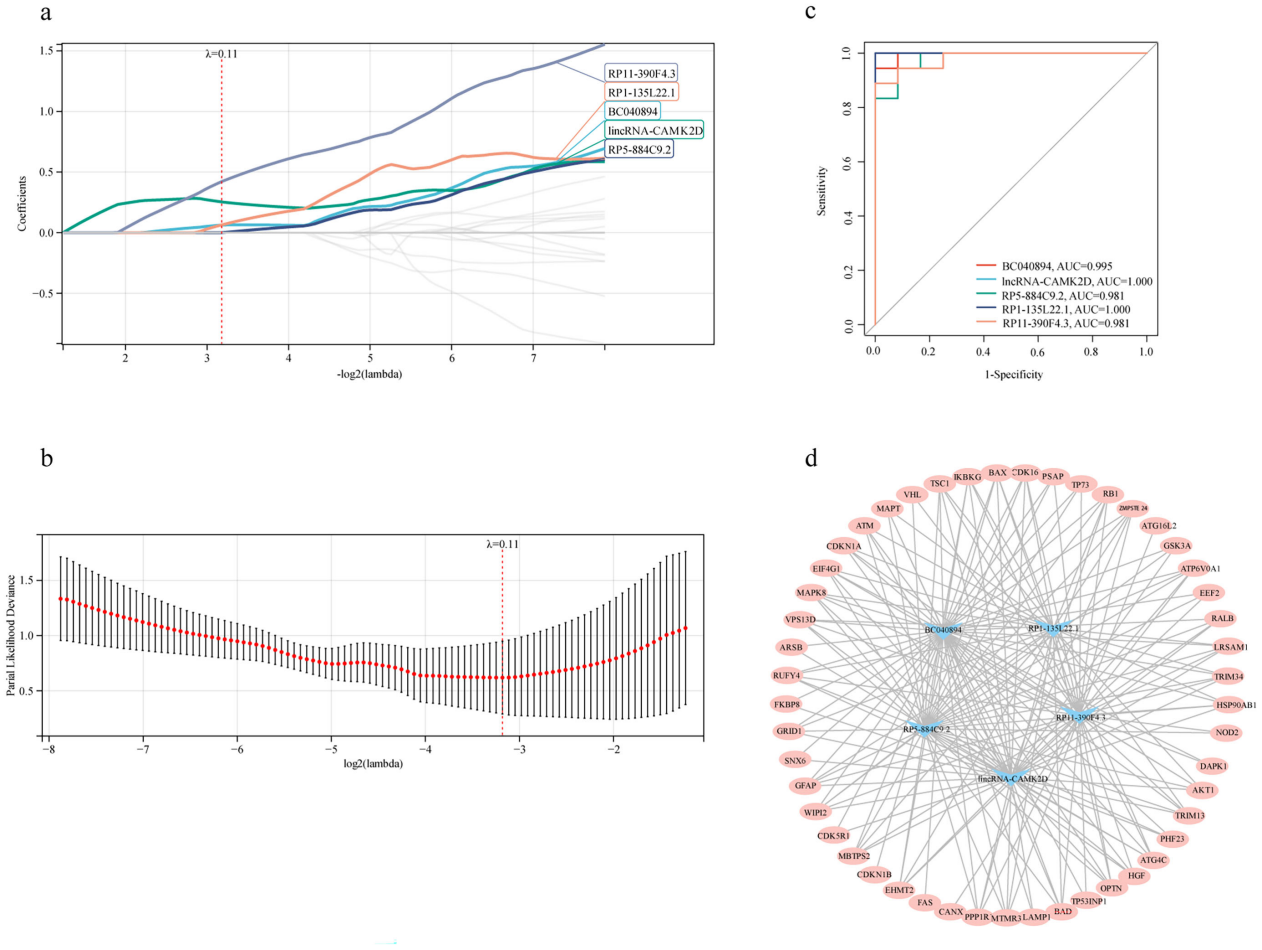
The co-expression network indicated the relationship between diagnostic autophagy-related lncRNAs and DEARGs. **(a, b)** LASSO Cox regression analysis for the diagnostic autophagy-related lncRNAs. **(c)** ROC curves of five diagnostic autophagy-related lncRNAs. **(d)** The co-expression network between diagnostic autophagy-related lncRNAs and DEARGs.

To identify lncRNAs associated with autophagy, a Spearman correlation analysis (with |r| > 0.7 and a *p*-value < 0.05) between core lncRNAs and DEARGs was performed, resulting in 365 differentially expressed autophagy-related lncRNAs. These 365 lncRNAs were then utilized for identifying candidate diagnostic autophagy-related lncRNAs by using LASSO Cox regression. We set the Lambda value to 0.110293666617702 and five lncRNAs (BC040894, lincRNA-CAMK2D, RP5-884C9.2, RP1-135L22.1 and RP11-390F4.3) were identified as potential diagnostic factors (**Figures 5a, b**). The area under the ROC curve was separately for BC040894 = 0.995, lincRNA-CAMK2D = 1.000, RP5-884C9.2 = 0.981, RP1-135L22.1 = 1.000, RP11-390F4.3 = 0.981 (**Figure 5c**). A co-expression network between 5 diagnostic autophagy-related lncRNAs and 49 DEARGs was built using Cytoscape software (**Figure 5d**).

### 3.5 Construction of a diagnostic lncRNA-mediated ceRNA network for EOS

**Figure 6:**
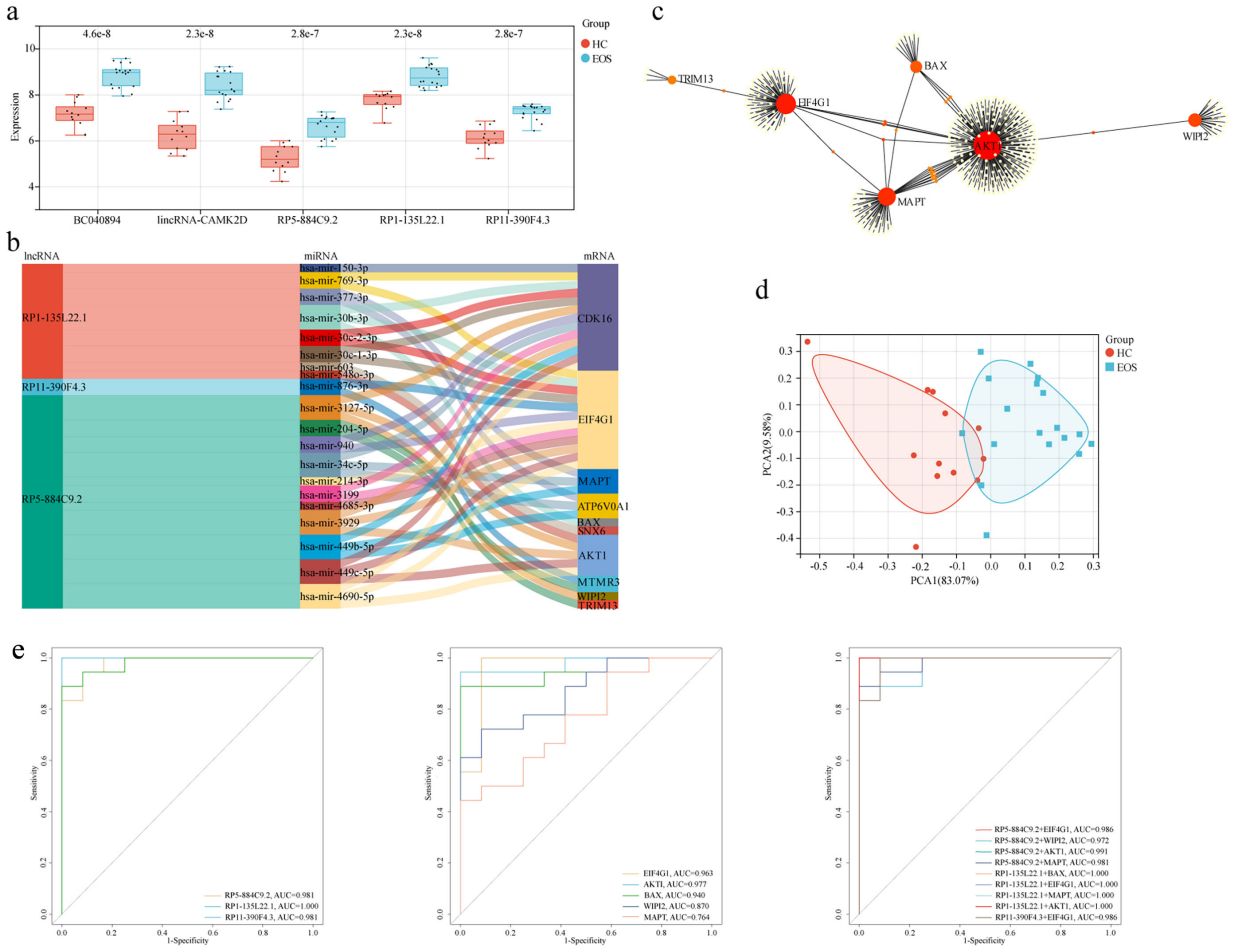
Construction of a diagnostic lncRNA-mediated ceRNA network and identification of hub mRNAs. **(a)** The box diagram showed the expression level differences of diagnostic autophagy-related lncRNAs between EOS and HC. **(b)** The Sankey diagram of the ceRNA network included three diagnostic autophagy-related lncRNAs, twenty predicted miRNAs, and ten DEARGs. **(c)** PPI network of DEARGs in the ceRNA network. **(d)** PCA of EOS and HC samples. **(e)** ROC curves of integrated lncRNAs-mRNAs, lncRNAs alone, and mRNAs alone.

We constructed an lncRNA-mediated ceRNA network to explore the regulatory mechanism between lncRNAs and mRNAs. Interestingly, the expression of all five diagnostic autophagy-related lncRNAs was significantly upregulated in EOS (**Figure 6a**). Based on the assumption of ceRNA, the expression of mRNAs should be positively correlated with lncRNAs. We selected 18 DEARGs upregulated in EOS (*EIF4G1*, *AKT1*, *ATP6V0A1*, *BAX*, *WIPI2*, *MTMR3*, *LRSAM1*, *TP73*, *ATM*, *TSC1*, *OPTN*, *VHL*, *TRIM13*, *CDK16*, *GFAP*, *TRIM34*, *MAPT*, *and SNX6*) (**Table 2**) for the construction of a ceRNA network. As described in the Methods section, the lncRNASNP2 database was used to screen the interaction between 4 diagnostic autophagy-related lncRNAs and 152 predicted miRNAs, and the TarBase database was used to identify 436 predicted miRNAs of 17 DEARGs. Finally, the miRNA-mRNA pairs and the lncRNA-miRNA pairs were integrated to construct a diagnostic lncRNA-miRNA-mRNA ceRNA network for EOS which was composed of three diagnostic autophagy-related lncRNAs (RP5-884C9.2, RP1-135L22.1, and RP11-390F4.3), twenty predicted miRNAs, and ten DEARGs (*AKT1*, *EIF4G1*, *MAPT*, *WIPI2*, *BAX*, *CDK16*, *ATP6V0A1*, *SNX6*, *MTMR3*, and *TRIM13*) (**Figure 6b**, **Table 3**).

**Table 2:** List of the main Gene abbreviation.

Gene abbreviation	full names of genes
*AKT1*	Serine/Threonine Kinase 1
*ATM*	AKT Serine/Threonine Kinase 1
*ATP6V0A1*	ATPase H+ Transporting V0 Subunit A1
*BAX*	BCL2 Associated X
*CDK16*	Cyclin Dependent Kinase 16
*EIF4G1*	Eukaryotic Translation Initiation Factor 4 Gamma 1
*GFAP*	Glial Fibrillary Acidic Protein
*LRSAM1*	Leucine Rich Repeat And Sterile Alpha Motif Containing 1
*MAPT*	Microtubule-associated protein tau
*MTMR3*	Myotubularin Related Protein 3
*OPTN*	Optineurin
*SNX6*	Sorting Nexin 6
*TP73*	Tumor Protein P73
*TRIM13*	Tripartite Motif Containing 13
*TRIM34*	Tripartite Motif Containing 34
*TSC1*	TSC Complex Subunit 1
*VHL*	Von Hippel-Lindau Tumor Suppressor
*WIPI2*	WD Repeat Domain, Phosphoinositide Interacting 2

**Table 3:** List of lncRNAs, miRNAs, and mRNAs in the ceRNA network.

lncRNA	miRNA	mRNA
RP1-135L22.1	hsa-mir-150-3p	*CDK16*
	hsa-mir-769-3p	*CDK16, EIF4G1*
	hsa-mir-377-3p	*MAPT, ATP6V0A1*
	hsa-mir-30b-3p	*BAX, CDK16, EIF4G1*
	hsa-mir-30c-2-3p	*CDK16, EIF4G1*
	hsa-mir-30c-1-3p	*CDK16, EIF4G1*
	hsa-mir-603	*SNX6*
RP11-390F4.3	hsa-mir-548o-3p	*AKT1*
RP5-884C9.2	hsa-mir-876-3p	*MTMR3, EIF4G1*
	hsa-mir-3127-5p	*WIPI2, AKT1, CDK16*
	hsa-mir-204-5p	*TRIM13, MTMR3*
	hsa-mir-940	*CDK16, EIF4G1*
	hsa-mir-34c-5p	*MAPT, ATP6V0A1, CDK16*
	hsa-mir-214-3p	*EIF4G1*
	hsa-mir-3199	*CDK16, EIF4G1*
	hsa-mir-4685-3p	*EIF4G1*
	hsa-mir-3929	*AKT1, CDK16, EIF4G1*
	hsa-mir-449b-5p	*MAPT, ATP6V0A1, CDK16*
	hsa-mir-449c-5p	*AKT1, CDK16, EIF4G1*
	hsa-mir-4690-5p	*AKT1, EIF4G1, CDK16*

### 3.6 Identification of hub mRNAs of the ceRNA network

A PPI network of DEARGs included in the ceRNA network was constructed using the STRING database (**Figure 6c**). We used degree ≥8 as the screening parameter and screened five hub proteins (including *AKT1*, *EIF4G1*, *MAPT*, *WIPI2*, and *BAX*). The PCA analysis indicated that these hub mRNAs can distinguish EOS patients from HC significantly (**Figure 6d**). The area under the ROC curve was separately for *EIF4G1 =* 0.963, *AKT1 =* 0.977, *BAX* = 0.940, *WIPI2 =* 0.870, *MAPT* = 0.764 (**Figure 6e**). Furthermore, ROC curve analysis demonstrated that integrating three diagnostic lncRNAs along with five hub mRNAs appeared to yield better diagnostic accuracy compared to using either lncRNAs or mRNAs alone.

### 3.7 Immune cell infiltration in EOS

**Figure 7:**
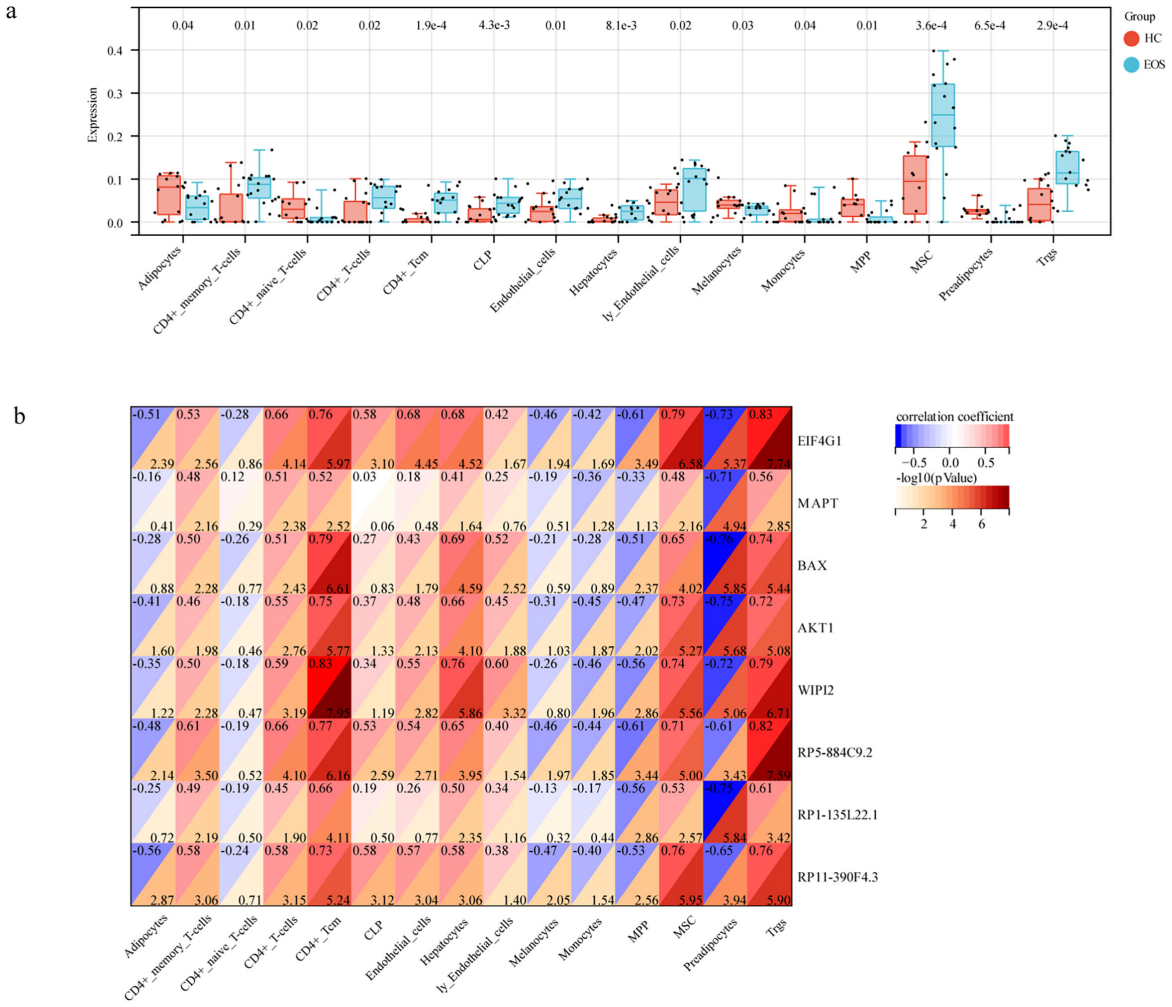
Immune cell infiltration in EOS. **(a)** The box diagram showed the differences in immune cell infiltrates between EOS and HC. **(b)** Correlation analysis between hub genes and immune cells.

We evaluated the differences in immune cell infiltration between EOS and HC by the xCell algorithm. As shown in **Figure 7a**, significant differences were demonstrated in the infiltration of 15 immune cell types such as CD4(+) memory T cells, CD4(+) naive T cells, and regulatory T cells (p < 0.05). It indicated that the infiltration of these immune cells may be closely related to the disease state. Through performing a correlation analysis between immune cell infiltration and lncRNAs/mRNAs expression levels, we found that all three diagnostic lncRNAs and five hub mRNAs were positively correlated with at least two types of immune infiltration (**Figure 7b**). This indicated that these autophagy-related ceRNAs may provide valuable information for guiding immunotherapy.

### 3.8 Conclusion

In conclusion, through transcriptome analysis, we systematically explored the autophagy-related ceRNA network associated with diagnosis and constructed a network related to the occurrence of EOS, which includes 3 lncRNAs, 20 miRNAs, and 10 mRNAs. Through PPI network analysis, we ultimately identified 5 key mRNAs that demonstrated good predictive ability in distinguishing EOS patients from HC. The combined use of 3 diagnostic lncRNAs and 5 key mRNAs appears to enhance diagnostic efficacy. Furthermore, all 3 diagnostic lncRNAs and 5 key mRNAs showed a positive correlation with at least two types of immune infiltration. We identified an autophagy-related ceRNA network for the early diagnosis of EOS, where the lncRNA-miRNA-mRNA network plays a role in the regulation of cellular autophagy, which may contribute to a deeper understanding of the pathogenesis of EOS.

## 4 Discussion

SCZ has a poor clinical course and outcome, partly due to a lack of effective early diagnosis. Unlike most diagnosable diseases, the diagnosis of SCZ is still based on clinical symptoms, which are subjective and variable, and may lead to delayed diagnosis or misdiagnosis (15). However, the prognosis of EOS which begins in childhood or adolescence is even worse. Therefore, searching for sensitive and specific biomarkers is meaningful for the early diagnosis and treatment of EOS. Increasing evidence has indicated that impaired autophagy is involved in the pathophysiology of psychiatric disorders such as SCZ (16, 17). We speculated that lncRNAs/mRNAs involved in the autophagy process may be potential diagnostic biomarkers or therapeutic targets for EOS.

In the study, a total of 365 core lncRNAs and 55 DEARGs were identified in EOS patients compared with HC. By implementing co-expression analysis and Lasso Cox regression, we identified five diagnostic autophagy-related lncRNAs (BC040894, lincRNA-CAMK2D, RP5-884C9.2, RP1-135L22.1, and RP11-390F4.3) and used ROC curve analysis to evaluate the predictive accuracy. By predicting the targeted miRNAs of autophagy-related lncRNAs and upregulated DEARGs, we constructed an autophagy-related lncRNA-miRNA-mRNA ceRNA network for the diagnosis of EOS which was composed of three diagnostic autophagy-related lncRNAs (RP1-135L22.1, RP5-884C9.2, and RP11-390F4.3), twenty predicted miRNAs, and ten DEARGs.

The functioning of lncRNAs as competitive endogenous RNAs mainly occurs in the cytoplasm (18). We used the lncRNA subcellular localization predictor (lncLocator) to understand the subcellular localization of these three lncRNAs. All of the lncRNAs were predicted to reside in the cytoplasm, including RP1-135L22.1 (53.82%), RP5-884C9.2 (35.68%), and RP11-390F4.3 (42.67%), suggesting that they were involved in the ceRNA regulatory network in the cytoplasm. Among them, two lncRNAs RP5-884C9.2 and RP1-135L22.1 have never been reported before. It was reported that RP11-390F4.3 is induced by hypoxia/HIF-1α and is involved in epithelial-mesenchymal transition and tumor metastasis (19). These three autophagy-related lncRNAs may be new biomarkers for EOS that have to be tested by further experiments.

Recent research findings support the important role of ARGs and pathways in SCZ. The research conducted by Lappas et al. found abnormal expression of ARGs in the hippocampus of patients with chronic SCZ, indicating that the autophagy pathway plays a crucial role in the pathological processes associated with SCZ (20). Bjornson et al. (21) emphasize the important role of ARGs’ activity in patients with SCZ (21). We further discovered five hub mRNAs of the ceRNA network (*EIF4G1*, *AKT1*, *MAPT*, *WIPI2*, and *BAX*) with good predictive accuracy of EOS, and the PCA analysis showed that these hub mRNAs can distinguish EOS patients from HC significantly. Eukaryotic Translation Initiation Factor 4 Gamma 1 (*EIF4G1*) is involved in the control process of translation which affects cellular survival and cellular death. The high levels of *EIF4G1* may increase cell proliferation and prevent autophagy (22). Some studies implied that *EIF4G1* may be related to EOS. Proteomic analysis revealed that *EIF4G1* was a potential target of Cullin 3 (*CUL3*) deficiency, a risk factor for autism spectrum disorder (ASD) and SCZ that caused cellular and behavioral defects. The level of *EIF4G1* was increased in CUL3-deficient brains (23). A study exploring peripheral blood miRNA biomarkers in patients with first-episode schizophrenia (FES) found that miR-4467 may be a non-invasive biomarker and *EIF4G1* was a hub target gene of miR-4467 (24). The research conducted by Tan et al. (25) underscores the critical role of ARGs in the onset and advancement of SCZ (25). Our results align with the conclusions drawn by Tan et al., particularly concerning the role of *EIF4G1*, which we similarly identified as a central mRNA within the ceRNA network. Tan et al. illustrated the substantial significance of *EIF4G1* in the context of SCZ, thereby corroborating our findings that underscore its potential as a diagnostic biomarker. Serine/Threonine Kinase 1 (*AKT1*) plays an indispensable role in autophagy. The physiological *AKT-MTORC1* signaling pathway is considered a key node in the regulation of macroautophagy/autophagy. Under nutrient-rich conditions, *AKT* can negatively regulate autophagy by activating the mechanistic target of rapamycin complex 1 (mTORC1), inhibiting the activity of the transcription factor forkhead box class O (FoxO), and inhibiting the expression of autophagy genes (26, 27). In current studies, the changing trend of *AKT1* expression in SCZ patients was inconsistent. A study found that the expression levels of *AKT1* significantly increased in acute schizophrenia patients (28), which is consistent with our result. Another case-control study showed significantly decreased expression of *AKT1* in male recent-onset SCZ patients (29). The results of this study were contrary to ours, possibly because the subjects were ethnically different, coming from the Netherlands and China respectively. BCL2 Associated X (*BAX*) is a pro-apoptotic gene that plays an indispensable role in regulating the mitochondrial apoptosis pathway. Recent studies have revealed cross-talk between the apoptotic and autophagic pathways, and many genes can play a role in both responses. *BAX* was identified as an important gene with the ability to induce mitochondrial autophagy in the process of apoptosis (30, 31). Evidence has indicated that the expression level of the *BAX* gene is upregulated in SCZ rats, which was consistent with our result (32). The upregulated *BAX* may be involved in apoptosis and autophagy processes in EOS. WD Repeat Domain, Phosphoinositide Interacting 2 (*WIPI2*), the mammalian homolog of the yeast *ATG18* gene, plays a critical role in autophagosome biogenesis (33). It was reported that *WIPI2* promoted mature autophagosome biogenesis, and when depleted, cells fail to form autophagosomes and engulfment of pathogenic bacteria (34). *WIPI2* has never been reported in SCZ before. The study found that the mutation of the *WIPI2* gene was associated with other neurodevelopmental diseases, such as global developmental abnormalities. Microtubule-associated protein tau (*MAPT*) encodes tau protein, the main component of neurofibrillary tangles (NFTs). *MAPT* protein aggregation inhibits the fusion of autophagosome and lysosome, resulting in autophagy disorders (35). *MAPT* has been reported to be associated with the onset of neuropsychiatric disorders, such as SCZ, Alzheimer’s disease, and Parkinson’s disease (36). Patients with SCZ have genetic variants, differential expression, and abnormal methylation in the *MAPT* gene that are linked with the disease risk (37, 38). A study has suggested the existence of tau-related neurodevelopmental disorders in adolescent psychosis (39). Therefore, these five hub genes may be effective biomarkers for the diagnosis of EOS.

Autophagy plays a critical role in the development, maintenance, and survival of various immune cell types, such as macrophages, neutrophils, T lymphocytes, and B lymphocytes (40). Dysregulation of autophagy can lead to impaired immune responses and contribute to the development of various diseases, including psychiatric disorders such as SCZ (41). In our study, immune cell infiltration analysis revealed significant differences in the infiltration of 15 immune cell types, such as CD4(+) memory T cells, CD4(+) naive T cells, and regulatory T cells between EOS patients and HC. Furthermore, all three diagnostic lncRNAs and five hub mRNAs were positively correlated with at least two types of immune infiltration. *EIF4G1* plays a critical role in the regulation of translation, which in turn influences cellular survival and the process of autophagy. Elevated levels of *EIF4G1* have the potential to enhance cellular proliferation while inhibiting autophagy, thereby possibly leading to immune dysregulation (42). *AKT1* is integral to the regulation of autophagy. The AKT-mTORC1 signaling pathway exerts a negative regulatory effect on autophagy in nutrient-abundant environments, thereby impacting the functionality and viability of immune cells (43). *BAX* is a pro-apoptotic gene that plays a critical role in the regulation of the mitochondrial apoptosis pathway. *BAX* is also implicated in mitochondrial autophagy, and its increased expression in patients with EOS may influence both apoptotic and autophagic processes, thereby affecting the functionality of immune cells (44). *WIPI2* plays a pivotal role in the biogenesis of autophagosomes, facilitating their maturation, which is essential for cellular responses to immunological challenges (34, 45). *MAPT* plays a role in the development of neurofibrillary tangles. The aggregation of *MAPT* proteins disrupts the fusion of autophagosomes and lysosomes, resulting in autophagy dysfunction and possibly affecting immune responses (46). These results highlight the crucial involvement of autophagy in the pathophysiological mechanisms underlying EOS and its possible interactions with the immune system.

However, this study has some limitations, which need further discussion in future work. First, the sample size in this study was relatively small, so the results obtained need further validation in a large sample. Second, the results obtained were only based on bioinformatics analytical methods, so we need to verify the findings through animal experiments and cellular experiments in future research. Finally, in this study, we utilized PBMC to analyze mRNA and lncRNA expression profiles. Although PBMC serve as a convenient and minimally invasive source for biomarker identification, they may not comprehensively represent the molecular alterations occurring within the brain. Therefore, future research should contemplate the incorporation of brain tissue samples or the application of advanced imaging methodologies to corroborate the findings observed in peripheral analyses. By addressing these limitations in subsequent research, we can enhance our comprehension of the role of autophagy in EOS and facilitate the advancement of precise diagnostic instruments and targeted therapeutic strategies. Notwithstanding these constraints, our study establishes a significant basis for investigating autophagy-related ceRNA networks as prospective biomarkers for the early diagnosis and treatment of EOS.

## Data Availability

The datasets presented in this study can be found in online repositories. The names of the repository/repositories and accession number(s) can be found below: https://www.ncbi.nlm.nih.gov/, GSE54913.
